# Civil Hospitals and Dispensaries of Bombay

**Published:** 1905-08-12

**Authors:** 


### CIVIL HOSPITALS AND DISPENSARIES OF BOMBAY.

On January 1st, 1903, there were 638 different institutions for the relief of the sick and suffering in the Presidency of Bombay. In the last day of that year 644 institutions were at work, 22 new ones having been opened during the 12 months and 16 closed. This is, on the whole, very satisfactory, because now in many localities European medical aid is thus brought within the reach of a vast number of the population, and the high statistics of attendance show that the privilege is appreciated. But there are large tracts still unprovided for, and in his report the Surgeon-General for the Government of Bombay expresses his regret that private enterprise does so little to meet the difficulty Numbers of students pass out yearly from the various schools and colleges, but they appear to settle down in the larger cities already well supplied with medical assistance, instead of in the rural districts where the services of a doctor are frequently so much needed, and where, in addition to the possibility of gaining a livelihood, an active and healthy life can be offered to the practitioners. The total number of patients treated during 1903 in the hospitals was 4,032,441 as compared with 4,031,185 in 1902, showing an increase of 1,256. Of these 67,066 were indoor and 3,965,375 outdoor patients. The ratio per cent. of males treated was 66 per cent., females 34 per cent. Malarial fever, as usual, accounted for the largest number of cases. Although a good deal has been effected in this direction by the aid of quinine, which is now sold cheaply at the post-offices, and is bought fairly freely, the large total of victims to malarial poison does not as yet show much decrease, and it is to be feared that the cases will always maintain a high average. Something can, of course, be done by attacking the mosquito in cantonments, but in the country this is more difficult. Tuberculosis, too, claims many victims, and it is found extremely difficult to persuade the people to take the proper steps to prevent the disease being spread. Cholera, on the contrary, has not been so prevalent during 1903. Improved sanitary surroundings and a better water supply have undoubtedly diminished the epidemics once so regular and constant a feature in the health of the people. Dysentery, diarrhœa, and diseases of the digestive system are accountable for a large number of admissions, doubtless the result of the past years of scarcity and privation, As matters improve with good and plentiful food, the diminution in the prevalence of these and other diseases may reasonably be expected. With regard to plague, the total number of patients admitted to the hospitals and afterwards found to be suffering from the disease was 1,197. The total number of cases reported in 1903, including the Native States, was 58,523, of whom 347,830 died. There was a very marked increase over the number treated in 1902.

The total number of operations performed in 1903 was 84,558, a decrease of 2,253 from the previous year. The decrease was amongst the minor operations, the major operations being slightly larger in number and quite equal in importance to those performed in the previous year. The Surgeon-General especially draws attention to the character of the operations for removal of vesical calculi, which included 948 litholapaxies, 446 lithotomies, and 95 lithotrites, and goes on to express regret that the number of cutting operations still remains so large. Whilst admitting that some were necessary, he thinks that the comparison between the crushing and the cutting operations points to a deficiency in the instruments for the former, and steps will be taken to supply lithotrites where they are required. Much has already been done with regard to instruments in the hospitals. Wooden-handled instruments are gradually being replaced by others which are capable of standing repeated sterilisation. This, added to the improvements in operation-rooms generally, reduces the danger of septic contamination to a minimum, and the general results of the operations performed attest the benefits already derived from these improvements. The total expenditure for the year was 14,17,089 rupees, the average cost of each patient being a fraction more than the year before.

